# How *Communications Psychology* editors evaluate your research

**DOI:** 10.1038/s44271-023-00014-z

**Published:** 2023-08-29

**Keywords:** Scientific community, Peer review, Psychology

## Abstract

Editorial work should not be a blackbox that leaves authors guessing for reasons. Here, we discuss what *Communications Psychology’s* evaluation criteria for research Articles are so authors can understand the decision-making surrounding when and why we send papers out for peer review.

One of the questions editors are most frequently asked is: how do you decide which paper to send to peer review? At *Communications Psychology*, all editors, that is in-house professional editors and editorial board members, use the same set of criteria to evaluate each manuscript.

**Figure:**
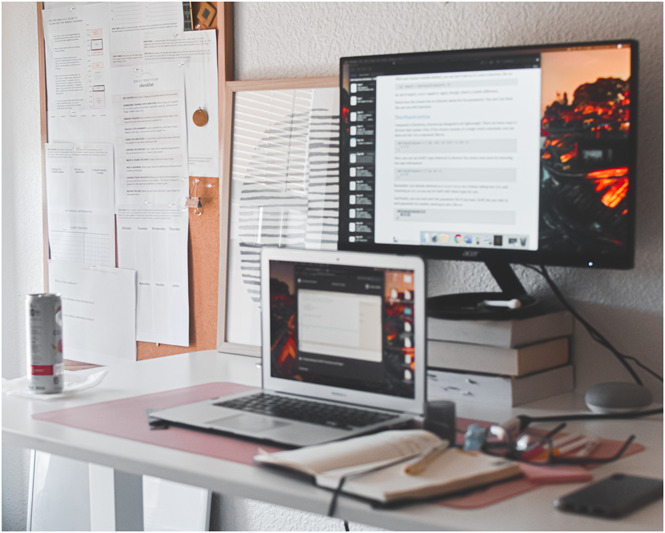
Photo by Hana on Unsplash

These criteria reflect the journal’s aims and values. For every manuscript, we make an assessment of the substance of the paper, the potential meaning to the field, and the paper’s potential to grow.

Some manuscripts are handled entirely by in-house editors, others are handled in close collaboration by an editorial board member and an in-house editor. When a paper is submitted, a primary handling editor (who may be an editorial board member) and one secondary handling editor (typically in-house) will look over the submission and decide whether to send it for peer review. They will then briefly discuss the paper, and consult over any difference of opinion.

## The meat of the paper

A key aspect of the first editorial read of each paper is to gauge how strongly the data support the conclusions. Various factors play into this judgment.

Editors make a high-level assessment of the general appropriateness of the methods for the research question. For example, would the approach to data collection and analysis be considered state-of-the-art; is it appropriate for the field, or could be sufficiently improved; or does it appear clearly sub-standard?

Relatedly, we consider whether the data seem to be of good quality, and if the work appears appropriately powered. Again, this judgment considers the present standard of research in the field and recent publications may be used as references to benchmark data quality.

In some domains of psychology, effect sizes clearly matter a lot to the relevance of a study. We appreciate that different types of research vary in the effect sizes that are meaningful to the field and make sure to take this into account in our assessment.

The journal cares about the strength of evidence in support of the conclusions. We assess whether the evidence appears strong, whether further support might be needed, or whether the conclusions are clearly not warranted. This includes an assessment of the statistical evidence; for example, studies where the key result is the absence of an effect or difference (“null-results studies”) are expected to demonstrate sufficient power to detect small effect sizes and substantive positive evidence for the null, rather than reporting a non-significant finding derived from null-hypothesis significance testing.

Finally, we look for evidence of robust scientific practice. For example, preregistration for confirmatory research is strongly encouraged (and expected for clinical trials). If the manuscript mentions preregistration, we make a brief assessment if the study appears to follow preregistration, or whether there are clear unjustified deviations.

These editorial judgments prior to review are inevitably only approximate assessments. If a manuscript is taken forward to peer review, we rely on our referees, who are experts in their field, to provide us with a more detailed evaluation of the technical aspects. We ask them to inform us in greater detail on whether a paper’s methods and results meet or exceed the standards of the field, or how the evidence could be strengthened.

## The meaning of the paper

When we assess how meaningful a contribution will be to specialists working in the field, we differentiate between original research questions and replication or generalization studies.

For original research questions (“novel” studies), we look at three key issues:

How big is the advance that the work represents? This assessment refers to the contribution that the paper makes to existing knowledge. We look at what is shown in the context of what was known before: does the paper constitute a breakthrough; an incremental advance; or is it entirely a confirmation of what had been established already?

We are also interested in how productive the topic is in general. By productive, we mean whether we see a lot of research being published on the same or related research questions; a steady (or increasing) flow of papers; or little work in the domain. The last of these categories is not necessarily negative, if an idea is very novel maybe the work in the field will just have started growing. To gauge productivity across different areas, we rely on a mixture of our expertise from daily submissions, what we learn at conferences and in other conversations with scientists, and information we derive from database searches. Editorial board members, who are active scientists, naturally also rely on their own knowledge of their field. Where previous articles appear, i.e., whether they were published in high impact factor journals, does not feed into this assessment.

A third consideration is how significant a contribution is to the field. Whether a small, incremental advance will make a difference will depend partly on how many different lines of research it informs. This third criterion sets the amount of what we learn (how big is the advance) in relation to the productivity of the topic (how much research activity is there on the same or related questions). This rough equation also considers the potential multidisciplinary interest and/or the potential to make an applied contribution. Impact does not equal potential citeability and our assessments refrain from speculations as to how many citations a paper may gather.

For replication studies, the advance naturally doesn’t lie in presenting a novel research question or approach. Instead, we assess how important and influential the target study is to the field. We consider what difference the paper has made at the time, whether it continues to affect thinking in the field, and whether the finding has been replicated already. Based on these considerations, we categorize target papers into highly influential, relevant to specialists, or (at this stage) of negligible influence. This then in turn informs our estimate of the broader relevance of the replication.

For generalization studies—which form a special category of replication studies—we ideally want to see generalization to human populations that are underserved in psychological research. The most valuable generalization studies will be large-scale studies that include participants and researchers from the Global South or otherwise underserved populations.

## The potential of a paper

*Communications Psychology* is a selective journal, but our selection process prioritizes robustness and interest to specialists in the field. Of course, we would not shut the door on a paper that presents an exceptionally novel finding of  the broadest appeal. But this doesn’t describe what we expect of submissions: we look for papers that pursue a research question that matters to the community and answer this research question with strong evidence. No manuscript arrives in perfect shape, and we always consider how much each research submission can be nurtured through peer review.

